# Synthesis of Alumina Nanoparticles Using Plasma-Induced Microbubbles

**DOI:** 10.3390/mi17050527

**Published:** 2026-04-26

**Authors:** Yuma Minami, Yuudai Aokusa, Nobutoshi Ota, Yu Yamashita, Yoko Yamanishi

**Affiliations:** Department of Mechanical Engineering, Kyushu University 744 Motooka, Nishi-ku, Fukuoka 819-0395, Japanota@bmf.mech.kyushu-u.ac.jp (N.O.);

**Keywords:** plasma-induced microbubbles, nanoparticle, alumina, phase control

## Abstract

This study investigates the selective synthesis of α- and γ-alumina nanoparticles using plasma-induced microbubbles. Although plasma-induced bubbles provide an effective reaction environment for the synthesis of nanomaterials, precise phase control remains challenging. Herein, we demonstrate that the modulation of the pulse off time regulates the thermal environment within the bubbles. Optical emission spectroscopy revealed that a shorter off time maintains a high electron temperature, indicating substantial heat accumulation. This high-energy state promotes the atomization of the precursor mist and the subsequent growth of molten droplets, providing sufficient activation energy for the formation of the thermodynamically stable α-phase. In contrast, a longer off time leads to the formation of a metastable γ-phase because of insufficient heating and rapid quenching. These findings prove that alumina nanoparticles with desired crystal phase and size can be synthesized by controlling the thermal energy inside the plasma-induced microbubbles.

## 1. Introduction

Aluminum oxide (alumina, Al_2_O_3_) is an indispensable engineering ceramic in modern industry, renowned for its superior mechanical strength, chemical stability, and electrical insulation properties [1]. In addition to the thermodynamically most stable α-phase, alumina exhibits several metastable polymorphs, such as the γ-, δ-, and θ-phases [2].

These crystalline phases possess distinct physicochemical properties, necessitating precise phase selection based on the intended application. For instance, γ-alumina, possessing a high specific surface area and pronounced acid–base properties, is widely utilized as a catalyst support and adsorbent [3,4]. Conversely, the stable α-alumina phase exhibits exceptionally high thermal conductivity (approximately 30 W m^−1^ K^−1^ for single crystals [5]), high hardness, and superior heat resistance compared to other phases. Consequently, there is a surging demand for α-alumina in applications such as cutting tools, wear-resistant coatings [6,7], and heat-dissipating insulating materials (thermal interface materials) for next-generation power devices [8,9]. Moreover, recent studies have expanded its application scope to the biomedical field, identifying α-alumina nanoparticles as potent antigen carriers for cancer vaccines because of their autophagy-inducing properties [10]. In addition, α-alumina has also been investigated for radiation-related applications, such as radiation-resistant materials and high-dose dosimetric ceramics [11,12].

However, the preparation of α-phase nanoparticles is particularly challenging. Previous studies have shown that in nanoparticles with large specific surface areas, surface energy becomes the dominant factor governing phase stability [13,14]. Unlike in bulk materials, the metastable γ-phase—which possesses lower surface energy than the α-phase—is thermodynamically more stable. This size effect, thermodynamically elucidated by McHale et al. [15], results in a drastic elevation of the phase transition temperature to the α-phase in nanoparticles, often requiring temperatures above 1100 °C.

In conventional calcination methods, such as the sol–gel process, such high temperatures inevitably induce severe sintering and necking among particles, complicating the preparation of spherical particles with excellent dispersibility [16]. At the same time, gas-phase quenching methods, such as radiofrequency thermal plasma quenching, are suitable for the synthesis of spherical particles; however, excessive cooling rates in these methods often lead to the freezing of metastable phases, complicating the formation of the equilibrium α-phase [13,15].

Solution plasma technology has been extensively investigated in diverse fields, including nanoparticle synthesis, surface modification, and the fabrication of carbon materials [17,18]. Given that solution plasma provides a high-energy reaction environment accompanied by a rapid cooling mechanism, the present study proposes a novel method for the synthesis of alumina nanoparticles that enables crystal phase control using plasma-induced microbubbles generated via the application of pulsed voltage. Herein, we first fundamentally investigate plasma, which is the thermal source for the reaction within plasma-induced microbubbles. Subsequently, we characterize the synthesized alumina nanoparticles, specifically determining their particle size and crystal phase.

## 2. Concept of Nanoparticle Synthesis

**Figure 1:**
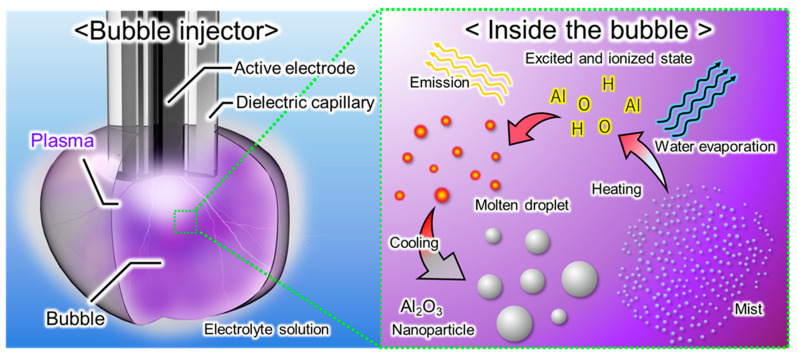
Schematic of the plasma-induced microbubble process. (**Left**): Overall experimental setup. A bubble injector is immersed in the electrolyte solution. Under applied voltage, a plasma-containing bubble is generated at the tip of the electrode. (**Right**): Internal reaction mechanism inside the bubble. The precursor solution exists as a mist in the gas phase. The mist is atomized by high-temperature plasma, followed by precursor decomposition to form molten alumina droplets. Finally, spherical nanoparticles are obtained via rapid cooling.

Figure 1 shows the conceptual framework of alumina nanoparticle synthesis using plasma-induced microbubbles. The experimental setup employs a specialized electrode, referred to as a “bubble injector.” This electrode has a core–shell structure consisting of a tungsten wire inserted into a ceramic tube, which is housed within a stainless steel tube. The entire electrode assembly is immersed in an aluminum nitrate aqueous solution serving as the electrolyte.

Upon the application of pulsed voltage, the generated electric field is concentrated at the tip of the electrode. This intense electric field initiates an electrical discharge within the internal gas phase, generating a plasma-induced microbubble [19,20].

Inside these plasma-induced microbubbles, it is considered that the aluminum nitrate precursor is predominantly transformed into alumina nanoparticles through a gas-phase thermal decomposition pathway [13], although possible minor contributions from the surrounding liquid phase cannot be completely excluded. The process proceeds via the following steps:

(1) $$2Al{\left(NO_{3}\right)}_{3}\cdot 9H_{2}O\to 2Al{\left(NO_{3}\right)}_{3}+18H_{2}O$$

(2) $$4Al{\left(NO_{3}\right)}_{3}\overset{∆}{\to }2Al_{2}O_{3}+12NO_{2}↑+3O_{2}↑$$

As the gas phase within the bubble is locally transformed into plasma, the interior of the bubble is presumed to be in a high-temperature state. Consequently, the surrounding aluminum nitrate solution is vaporized and dispersed as a mist in the bubble. Around the discharge electrode, where plasma is generated, the water in this mist evaporates, resulting in the concentration and precipitation of aluminum nitrate, as expressed in Equation (1). In the plasma region, the concentrated nitrate species can be excited and partially ionized, and may undergo fragmentation prior to thermal decomposition. Subsequently, as shown in Equation (2), molten aluminum nitrate thermally decomposes into alumina, which then solidifies into nanoparticles during cooling.

## 3. Materials and Methods

### 3.1. Experimental Setup

**Figure 2:**
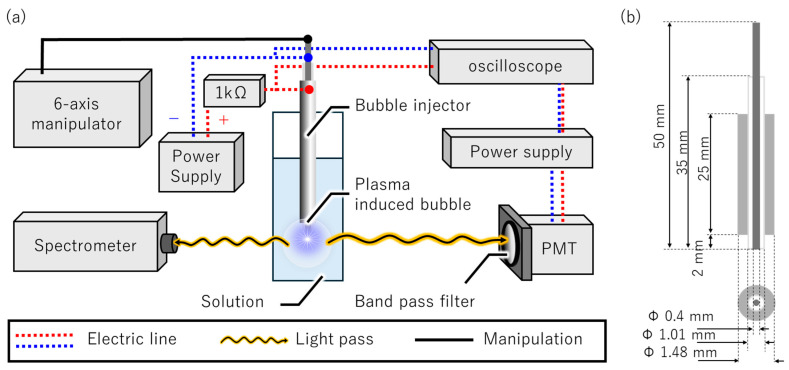
(**a**) Schematic of the experimental setup. (**b**) Structure and dimensions of the bubble injector.

Figure 2a shows the schematic of the experimental setup. The apparatus comprises a bubble injector, a bipolar pulsed power supply (KYL-1200, BEX, Tokyo, Japan), a 1-kΩ ballast resistor, and an oscilloscope (DS5614A, Iwatsu Electric, Tokyo, Japan). Optical emission measurements were conducted using a photomultiplier tube (PMT) module (H14603-20, Hamamatsu Photonics, Shizuoka, Japan) equipped with a dedicated power supply (C10709, Hamamatsu Photonics, Shizuoka, Japan) and a band pass filter (center wavelength: 656.27 nm), as well as a compact high-sensitivity 2D CCD spectrometer (Solid Lambda CCD, Spectra Co-op, Tokyo, Japan).

Figure 2b shows the structure and dimensions of the bubble injector. The bubble injector was fabricated by inserting an alumina ceramic tube (outer diameter: 1.0 mm, inner diameter: 0.5 mm, length: 35 mm) into a stainless steel tube (outer diameter: 1.48 mm, inner diameter: 1.30 mm, length: 25 mm), which served as the anode. A tungsten wire with a diameter of 0.4 mm, serving as the power cathode, was subsequently inserted into the alumina tube. An aqueous solution of aluminum nitrate nonahydrate (Al(NO_3_)_3_·9H_2_O) with a concentration of 1.5 mol/L was employed as the electrolyte.

### 3.2. Evaluation of Plasma Stability

First, the conditions required for stable plasma generation were investigated by varying the power supply parameters. To evaluate the discharge stability, we examined the dielectric breakdown behavior during the application of 100 pulses by simultaneously monitoring the voltage measured across the bubble injector and the output voltage corresponding to the H-α line (656.28 nm) emission from the plasma-induced microbubbles, which was selectively detected through a band pass filter (center wavelength: 656.27 nm) using the PMT module and recorded on the oscilloscope. In this experiment, the pulse width was varied from 5–20 µs, and the off time (the interval between two pulses) was varied from 5–100 µs in increments of 5 µs. The applied voltage was adjusted from 500–1000 V in 100-V increments. The total number of applied pulses was fixed at 100 for all experiments in this evaluation.

### 3.3. Evaluation of Plasma Emission Duration

Plasma acts as the thermal source driving the reaction; thus, the duration of plasma existence is a critical indicator directly linked to the effective reaction time. Therefore, we measured the duration of plasma emission per pulse. The H-α line generated at the 100th, 500th, and 1000th pulses during a 1000-pulse sequence was detected using the PMT. The duration of plasma emission was defined as the time during which the PMT output voltage exceeded 0.1 V. This threshold of 0.1 V was determined to be sufficiently higher than the background noise level observed in the absence of light emission. In this experiment, the off time was varied from 10–50 µs. The applied voltage, pulse width, and total number of pulses were fixed at 1000 V, 10 µs, and 1000 pulses, respectively.

### 3.4. Evaluation of the Electron Temperature

To evaluate the energy possessed by the plasma as a thermal source, the electron temperature of the plasma was calculated. Assuming that the plasma is in a state of local thermodynamic equilibrium, the electron temperature can be estimated from the intensity ratio of two spectral lines [21]. In the present study, the calculated values are used mainly as comparative indicators under different pulse conditions, because effects such as self-absorption, line broadening, and wavelength-dependent instrumental sensitivity were not rigorously corrected. The experimental parameters were set as follows: the pulse width was varied from 10–25 µs, and the off time was varied from 10–30 µs. The applied voltage and the total number of pulses were fixed at 1000 V and 1000, respectively. The wavelengths of the H-α (656.28 nm) and H-β (486.13 nm) lines were utilized for this calculation.

(3) $$T_{e}=\frac{E_{i}-E_{k}}{k_{b}In(\frac{I_{kl}\lambda_{kl}A_{ig}g_{ij}}{I_{ij}\lambda_{ij}A_{kl}g_{kl}})}$$

where *I*, *λ*, *g*, *A*, and *E* are the emission intensity, wavelength, statistical weight of the upper excited state, Einstein transition probability, and energy of the upper state, respectively. The subscripts *ij* and *kl* denote the transitions from the upper excited states *i* and *k* to their respective lower excited states, and *k_b_* is the Boltzmann constant.

### 3.5. Characterization of the Synthesized Nanoparticles

The size distribution and crystal phase of the synthesized nanoparticles were characterized. The particle size distribution was evaluated using scanning electron microscopy (SEM; JSM-IT700HR, JEOL, Tokyo, Japan). The projected area of each particle was measured from the SEM images and converted into the equivalent circular diameter. To ensure statistical reliability, more than 400 particles with equivalent circular diameters ranging from 15–300 nm were analyzed. The lower limit of 15 nm was set based on the resolution limit of the SEM instrument.

The crystal phase of the nanoparticles was identified using X-ray diffraction (XRD; SmartLab, Rigaku, Tokyo, Japan). The XRD patterns were recorded in the 2θ range of 15–75°. For these characterizations, samples synthesized under the following conditions were used: the off time was 10, 30, and 50 µs, whereas the pulse width was fixed at 10 µs. The applied voltage and the total number of pulses were fixed at 1000 V and 1000 pulses, respectively. Considering the sample preparation for these measurements, the solution treated with plasma was centrifuged to collect the particles. The collected precipitates were then washed with ultrapure water and vacuum-dried to recover the powders from the liquid phase.

## 4. Results

### 4.1. Evaluation of Plasma Stability

**Figure 3:**
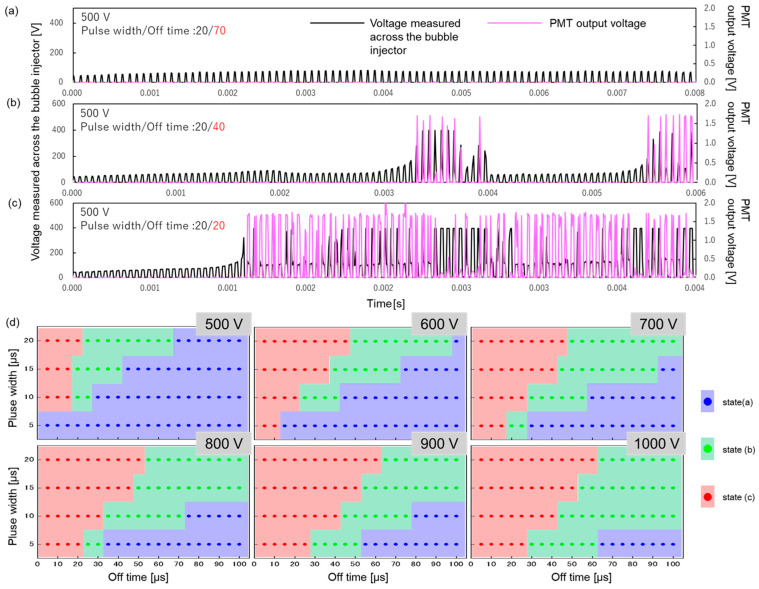
Waveforms of the voltage measured across the bubble injector and PMT output voltage. (**a**) No plasma breakdown is observed, resulting in no PMT output (state (a)). (**b**) Plasma breakdown occurs intermittently (state (b)). (**c**) Plasma breakdown occurs continuously, leading to continuous PMT output (state (c)). (**d**) Maps of discharge states based on applied voltage, pulse width, and off time.

Discharge behavior was classified into three modes, as defined in the experimental section. Figure 3 shows the typical signal waveforms corresponding to these three classifications. In the case of Figure 3a, no output signal is detected by the PMT, indicating that plasma emission did not occur (no discharge). In Figure 3b, PMT output signals are sporadically observed, revealing that dielectric breakdown occurred intermittently within the pulse sequence (intermittent discharge). In contrast, Figure 3c shows a consistent PMT signal for almost all applied pulses, confirming that plasma emission occurred continuously (continuous discharge). Figure 3d presents the mapping of these three discharge modes as a function of applied voltage, pulse width, and off time. With increasing applied voltage, the boundaries between the discharge modes shift toward longer off times. Considering temporal parameters, the discharge tended to transition to the continuous mode (state (c)) with increasing pulse width and decreasing off time. Based on these results, an applied voltage of 1000 V was adopted for subsequent experiments, as this condition was confirmed to ensure the most stable plasma generation.

### 4.2. Evaluation of Plasma Emission Duration

**Figure 4:**
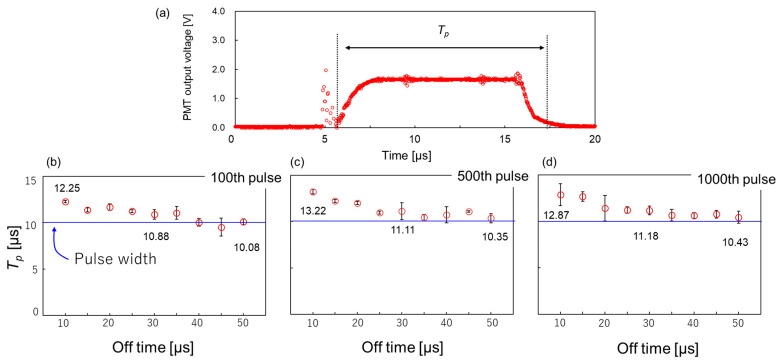
(**a**) PMT output waveform and definition of plasma emission duration (*T_p_*). (**b**) *T_p_* as a function of the off time at the 100th pulse. (**c**) *T_p_* at the 500th pulse. (**d**) *T_p_* at the 1000th pulse. Each experiment was repeated three times (*n* = 3).

Figure 4a shows a typical output voltage waveform obtained from the PMT for a single pulse. The duration during which this output voltage exceeds 0.1 V is defined as the plasma emission duration (*T_p_*). Electromagnetic noise unrelated to plasma emission is observed in the output signal at the rising edge of the applied pulse voltage. Therefore, this noise component was excluded from the determination of *T_p_*. Figure 4b presents the *T_p_* measured at the 100th, 500th, and 1000th pulses as a function of the off time. The plasma emission duration varies depending on the off time, even at a constant pulse width. Specifically, the emission duration tends to decrease with the extension of the off time. Under all experimental conditions, the emission duration exceeds the applied pulse width (10 µs), with recorded values ranging from 10.08 to 13.22 µs. Furthermore, as shown in Figure 4b–d, the 100th, 500th, and 1000th pulses do not notably differ in emission duration within the 1000-pulse sequence. This suggests that the number of applied pulses does not considerably affect the duration of plasma emission. Overall, these results indicate that the duration of plasma emission can be effectively controlled by modulating the off time.

### 4.3. Evaluation of the Electron Temperature

**Figure 5:**
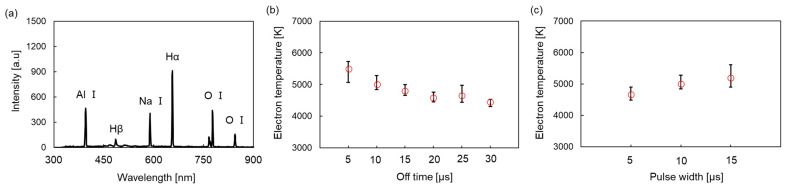
(**a**) Optical emission spectrum of plasma-induced microbubbles with atomic line assignments. (**b**) Electron temperature as a function of the off time. (**c**) Electron temperature as a function of pulse width. Experiment (**b**,**c**) was repeated five times (*n* = 5).

Figure 5a shows the optical emission spectrum of the plasma generated from the solution. In the spectrum, emission lines corresponding to the Balmer series of hydrogen (H-α at 656.28 nm and H-β at 486.13 nm), atomic oxygen (O-I at 777 and 844 nm), neutral aluminum (Al I at 394.4 nm), and neutral sodium (Na I at 589.0 nm) are clearly identified [22]. The electron temperature was calculated using the intensities of the H-α and H-β lines from these spectra via Equation (3). Figure 5b presents the electron temperature as a function of the off time. The electron temperature decreases with the extension of the off time. Figure 5c shows the dependence of the electron temperature on the pulse width. The results indicate that the electron temperature increases with the extension of pulse width.

### 4.4. Characterization of the Synthesized Nanoparticles

**Figure 6:**
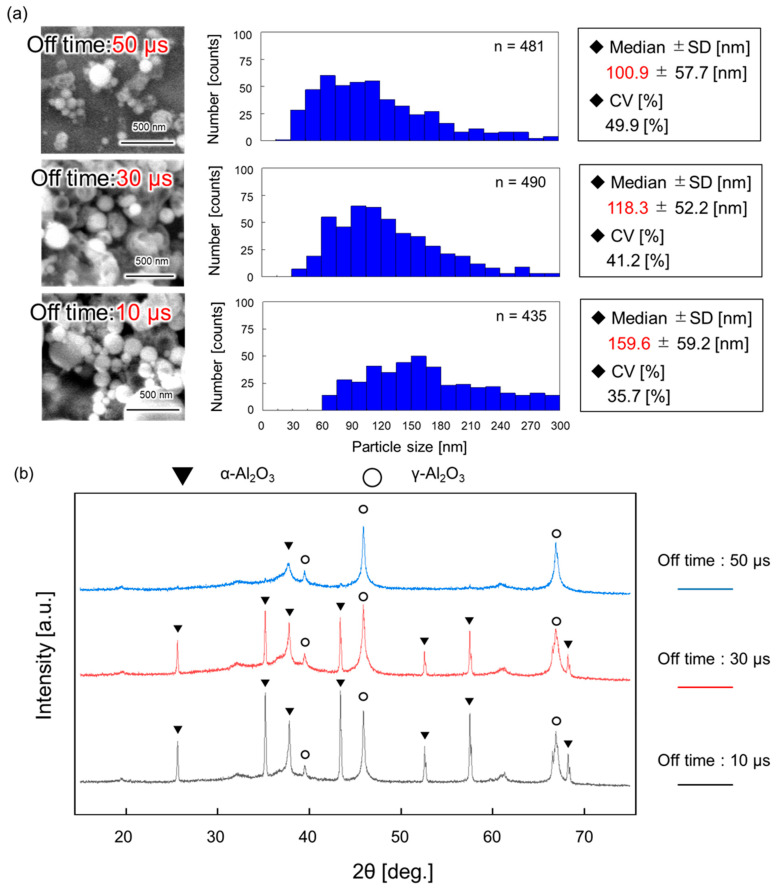
(**a**) Particle size distributions obtained at off times of 10, 30, and 50 µs. (**b**) XRD patterns of the synthesized nanoparticles. XRD: X-ray diffraction.

Figure 6a shows the size distribution of the synthesized nanoparticles. As evident from the graph, the particle size increases with the shortening of the off time. Specifically, the median diameter is 159.6 nm at an off time of 10 µs and 118.3 nm at 30 µs. The smallest particle size is observed at an off time of 50 µs, with a median diameter of 100.9 nm.

Figure 6b presents the XRD patterns of the obtained nanoparticles. The formation of alumina was confirmed under all experimental conditions. The γ-phase becomes dominant with the extension of the off time, whereas the α-phase becomes dominant with the shortening of the off time. These results indicate that the crystal phase of alumina nanoparticles can be controlled solely by adjusting the power supply parameters in this method.

## 5. Discussion

Herein, we showed that the crystal phase and size of alumina nanoparticles can be selectively controlled solely by modulating the pulse off time. Shortening the off time induced the formation of the thermodynamically stable α-phase, whereas its extension facilitated the formation of smaller γ-phase nanoparticles.

Synthesis was conducted under high voltage and short off time, where continuous plasma generation was confirmed (Figure 3c), as high temperatures are required for the synthesis of crystalline alumina. The trend in particle size shown in Figure 6a—where the size increases with the shortening of the off time—strongly correlates with the plasma emission duration (reaction time; Figure 4b). This suggests that the plasma emission duration represents the effective reaction time within plasma-induced microbubbles. Therefore, reaction time can be tuned by modulating the off time, enabling precise control over the particle size. Considering the crystal phase, a distinct dependence on the off time was also observed. The formation of α-phase-dominant alumina at short off times and γ-phase-dominant alumina at long off times is consistent with the trend in electron temperature shown in Figure 5b. This indicates that conditions with higher plasma energy (higher electron temperature) promote the formation of the stable α-phase, whereas conditions with lower energy favor the metastable γ-phase. Although particle formation in the surrounding liquid phase cannot be completely excluded, the spherical particle morphology and the observation of Al emission from the plasma-induced microbubble support the view that the bubble interior is the dominant formation site under the present conditions. These findings suggest that, within the present experimental range, off time control can be used to selectively control both the crystal phase and particle size. In a previous related study on plasma-induced bubbles, bubble behavior was also reported to vary with pulse off time, although bubble size and shape were not systematically evaluated in the present study [20]. However, the results also reveal a trade-off between the α-phase formation and size reduction: larger particles tend to be α-phase, whereas smaller particles tend to be γ-phase. The synthesis of smaller α-alumina nanoparticles requires reducing the growth time by shortening the pulse width. Although this would likely cause a decrease in particle size, it might also result in insufficient energy for the transition to the α-phase.

To overcome this limitation and synthesize small α-alumina nanoparticles at lower energies, the addition of seed materials (α-alumina particles) into the solution should be effective from both thermodynamic and kinetic perspectives. The promotion of crystal growth (homoepitaxial growth) on seeds with a matching atomic structure substantially lowers the interfacial energy barrier required for α-phase nucleation, thereby enabling its synthesis at lower energies than typically required [16,23].

## 6. Conclusions

Herein, we proposed a method for the selective synthesis of alumina nanoparticles with specific phase and size by modulating the pulse off time in a process utilizing plasma-induced microbubbles. Optical emission spectroscopy clearly revealed Al atomic lines alongside water decomposition species, suggesting that the synthesis proceeds via the gas-phase atomization of the precursor within the microbubbles. In the present study, off time was identified as a main practical control parameter strongly correlated with particle properties, likely through its influence on the thermal state and reaction duration within the bubbles. A short off time (10 µs) facilitated the formation of the thermodynamically stable α-phase because of the high thermal energy provided by the plasma, whereas a long off time (50 µs) resulted in the selective formation of finer metastable γ-phase particles driven by the enhanced quenching effect. Consequently, we showed that the material properties can be tuned solely by adjusting the electrical parameters within a single experimental system. Therefore, the proposed method is an effective on-demand process for the synthesis of functional oxide nanoparticles tailored for diverse applications, ranging from catalysts to thermal management materials. A practical advantage of this method is its simple experimental configuration, using only a pulsed power supply, a bubble-injector electrode, and a precursor solution containing aluminum ions. In addition, the liquid-phase environment may offer flexibility in precursor selection and could allow future chemical control through solution design. Although the present results show a clear correlation with off time, the possible contributions of other related factors, such as duty cycle, accumulated heating, and bubble behavior, should be examined in future work.

## Data Availability

All data generated or analyzed during this study are included in this published article and its Supplementary Information Files.

## Supplementary Materials

The following supporting information can be downloaded at https://www.mdpi.com/article/10.3390/mi17050527/s1.
