# Supplementary material for: Synthesis of Alumina Nanoparticles Using Plasma-Induced Microbubbles
Source: Micromachines (Basel). 2026 Apr 26;17(5):527. doi: 10.3390/mi17050527 (PMC13209001; doi:10.3390/mi17050527)
Supplement: Supplementary file 1 [file micromachines-17-00527-s001.zip › micromachines-4244187-supplementary.pdf]

Supplementally File

To assess the possible effect of the dielectric material of the bubble injector on nanoparticle formation, comparative experiments were performed using ceramic and glass tubes as the insulating component. Figure S1 summarizes the XRD results obtained with the two dielectric materials, showing no substantial difference in the obtained products under the present experimental conditions. In addition, Figure S2 presents the optical emission spectra measured for both cases, confirming that aluminum emission lines were observed irrespective of whether the insulating tube was ceramic or glass.

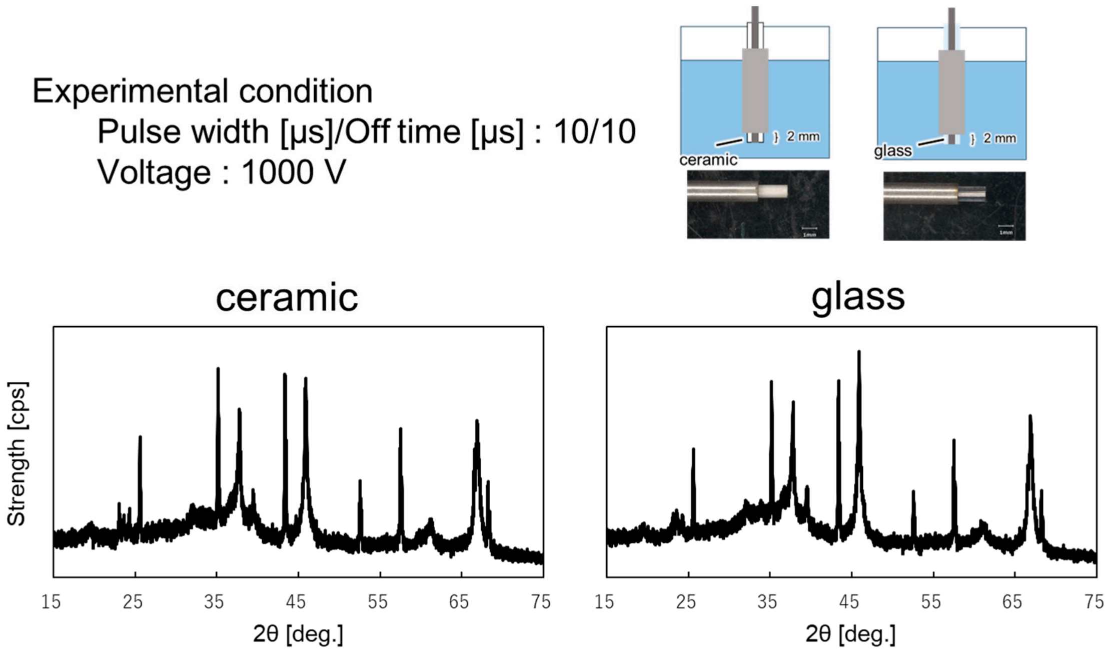

Figure S1 The XRD results obtained with the two dielectric materials

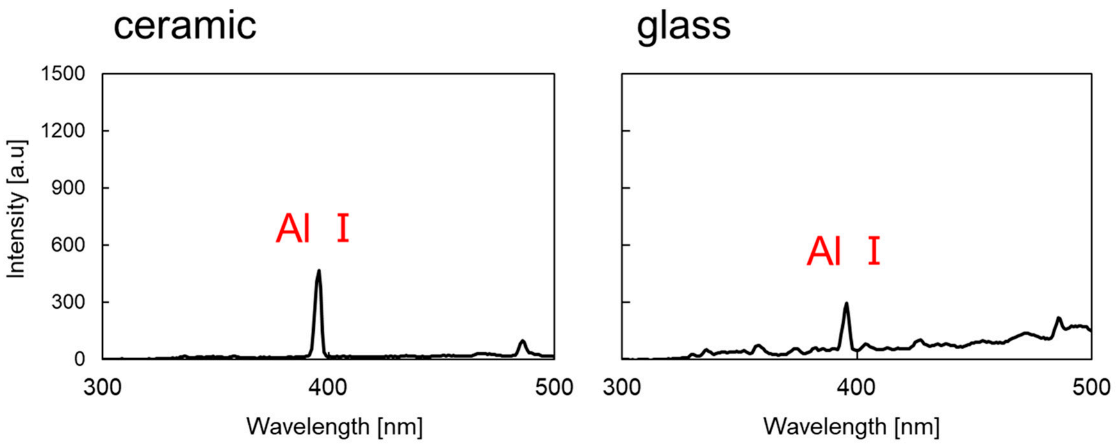

Figure S2 The optical emission spectra measured
